# Making America Great Again? National Nostalgia's Effect on Outgroup Perceptions

**DOI:** 10.3389/fpsyg.2021.555667

**Published:** 2021-04-14

**Authors:** Anna Maria C. Behler, Athena Cairo, Jeffrey D. Green, Calvin Hall

**Affiliations:** ^1^Psychology Department, North Carolina State University, Raleigh, NC, United States; ^2^Psychology Department, Virginia Commonwealth University, Richmond, VA, United States

**Keywords:** national nostalgia, prejudice, intergroup relations, emotion, political differences

## Abstract

Nostalgia is a fond longing for the past that has been shown to increase feelings of meaning, social connectedness, and self-continuity. Although nostalgia for personal memories provides intra- and interpersonal benefits, there may be negative consequences of group-based nostalgia on the perception and acceptance of others. The presented research examined national nostalgia (a form of collective nostalgia), and its effects on group identification and political attitudes in the United States. In a sample of US voters (*N* = 252), tendencies to feel personal and national nostalgia are associated with markedly different emotional and attitudinal profiles. Higher levels of national nostalgia predicted both positive attitudes toward President Trump and racial prejudice, though there was no evidence of such relationships with personal nostalgia. National nostalgia most strongly predicted positive attitudes toward president Trump among those high in racial prejudice. Furthermore, nostalgia's positive relationship with racial prejudice was partially mediated by perceived outgroup threat. Results from this study will help us better understand how the experience of national nostalgia can influence attitudes and motivate political behavior.

Throughout Donald Trump's tumultuous presidential campaign and tenure, journalists and scholars sought to explain his appeal to many American voters. In the 2016 presidential election, as many as nine million voters who previously supported Barack Obama, the first Black president, voted for Trump despite his inflammatory race-focused rhetoric (Skelley, 2017). One concept repeatedly emerged within these discussions as a mainstay of Trump's political appeal: that of *nostalgia*, broadly defined as a bittersweet longing for the past. Evidence of Trump's appeals to an earlier time in American history have been cited from the beginning of the 2016 presidential campaign through his failed 2020 reelection campaign, ranging from the salient nostalgic reverie of the “Make America Great Again” campaign slogan (Samuelson, 2016) to more coded political rhetoric promising White, working class Americans a return to times that have been lost (Brownstein, 2016).

Some have hypothesized that such nostalgic rhetoric may capitalize on voters' latent feelings of threat to their economic welfare, or to the racial or cultural homogeneity of American culture (Brownstein, 2016; Smeekes et al., 2020). On a broad scale, nostalgia focused on nationality is a prominent feature of right-wing populist party rhetoric, and evidence from voters in the Netherlands suggests that the emphasis of stigmatizing outgroups and preserving cultural hegemony within nostalgic messaging is what explains the link between nostalgia and right-wing populist support (Smeekes et al., 2020). In the United States, several studies provide strong evidence of a link between support for Trump and group prejudice. For example, survey research has indicated that racial and anti-immigrant resentment strongly predicted voters' support of Trump in 2016, more so even than voter's feelings of economic threat (Hooghe and Dassonneville, 2018; Mutz, 2018; Schaffner et al., 2018). Additionally, a longitudinal analysis of police reports evidenced a significant increase in hate crimes reported in Trump-supporting counties in the 6 months following the 2016 presidential election (Edwards and Rushin, 2018). However, no research has of yet established whether Trump's nostalgic rhetoric may be associated with voters' attitudes toward racial outgroups. To this end, in this paper, we present evidence that national nostalgia, an emotion distinct from personal nostalgia, is associated with increased prejudice as well as support for the populist messaging of Donald Trump.

## The Sociality of Nostalgia

Nostalgia is a mostly positive emotion that increases self-regard, attenuates self-esteem defense, enhances meaning in life, increases perceptions of self-continuity, and lessens feelings of existential threat (Wildschut et al., 2006; Routledge et al., 2008). Most people report experiencing nostalgia on a regular basis (Wildschut et al., 2006) and often structure their present in anticipation of experiencing nostalgia in the future (Cheung et al., 2020). Nostalgia is triggered in various ways, including by music, scents, and reflecting on past momentous events (Barrett et al., 2010; Reid et al., 2015; Sedikides et al., 2015b). This emotion also serves vital relational functions, increasing social connectedness and perceived social support (Sedikides et al., 2008).

The social connectedness function of nostalgia is a primary avenue through which nostalgia confers positive psychological benefits. Although nostalgic memories are more likely to be evoked while experiencing negative affect (Wildschut et al., 2006) and loneliness (Zhou et al., 2008), the content of nostalgic memories evoked during these emotional states seem to act as a “repository” of positive affect, positive self-regard, and social connectedness (Sedikides et al., 2008, p. 306). The content of nostalgic memories is predominantly social, including recollections of close others, important social events, or tangible objects reminiscent of loved ones (Wildschut et al., 2006; Batcho et al., 2008). As a result of this, nostalgic memories seem to indirectly regulate these positive emotions by evoking and making more salient one's symbolic connections with others (Sedikides and Wildschut, 2019). For example, nostalgia felt in response to loneliness has been shown to reduce perceptions of isolation and low social support (Zhou et al., 2008). In organizational contexts, nostalgic emotions buffer the negative effects of low social support (due to procedural injustice) on reduced cooperation (van Dijke et al., 2015).

Importantly, those who are more likely to experience nostalgia (i.e., those high in personal nostalgia) are also more motivated to control prejudicial feelings and reduce their expression of prejudices against outgroups as a result of these positive benefits (Cheung et al., 2017). Four studies of Caucasian Americans examined the links between personal nostalgia and the expression of both blatant and more subtle prejudice toward African Americans (Cheung et al., 2017). They found that the link between personal nostalgia and prejudice reduction was mediated by feelings of empathy, suggesting that the experience of nostalgia offers advantages beyond the self.

## National Nostalgia vs. Personal Nostalgia

The link between nostalgia and sociality becomes more complex when considering nostalgia felt for one's group. Although nostalgia felt at the individual level confers both intra- and interpersonal benefits, group-based nostalgia appears to have a distinct psychological profile from personal nostalgia. Group-based emotions, as distinct from individual-level emotions, arise when individuals self-categorize with a social group and integrate the group into their sense of self (Seger et al., 2009). Furthermore, group-based emotions can differ markedly from their analogous individual level counterparts, such as when an individual might feel strong pride and happiness for their home team while not feeling strong pride in themselves (Smith and Mackie, 2016). Furthermore, group-based emotions serve a regulatory function of strengthening positive attitudes and behavioral intentions toward both their ingroup and threatening outgroups (Smith et al., 2007; Seate and Mastro, 2015).

Group-based nostalgia—operationalized as nostalgia felt for events shared with one's ingroup, or *collective nostalgia*—can be experienced in a variety of social settings, including organizations, school classes (e.g., Class of 2021), cities, and nations (Wildschut et al., 2014; Smeekes, 2015; Green et al., 2021). Like individual-level nostalgia, shared memories can include notable events, such as a special performance (band or orchestra), graduation day, homecoming (college class), or sports championships (city). However, unlike individual-level nostalgia, group-based nostalgia can occur in the form of a longing for a past that individuals themselves did not experience, but rather one that was passed down through collective memory (Martinovic et al., 2017). Additionally, collective nostalgia has been shown to increase positive attitudes as well as an approach-oriented action tendency toward the ingroup relative to an individually experienced nostalgic memory (Wildschut et al., 2014, Study 1). Collective nostalgia also can increase group-oriented prosociality (e.g., willingness to volunteer or donate money to help the ingroup; Wildschut et al., 2014; Green et al., 2021). Collective self-esteem mediated this effect: recalling a collective nostalgic event increased collective self-esteem, which, in turn, increased intentions to volunteer. Other research has found additional ingroup benefits to collective nostalgia, such a preference for domestic (vs. foreign) consumer products (Dimitriadou et al., 2019) and a promotion of collective political action (in Hong Kong; Cheung et al., 2017).

However, there are two sides to this coin. A preference for domestic products is also a bias against foreign products, and the promotion of collective political action was driven by anger and contempt for the outgroup (i.e., Hong Kong residents toward mainland Chinese; Cheung et al., 2017). Individuals who recalled a collective nostalgic memory (vs. an ordinary collective memory) were more willing to punish outgroup members who were unfair to an ingroup member (Wildschut et al., 2014, Study 3). However, in some cases, collective nostalgia might increase intergroup contact when individuals can feel collective nostalgia for a *superordinate* group (Martinovic et al., 2017). In a study of former Yugoslavians who had settled in Australia, Bosniaks, Croats, and Serbs who identified with Yugoslavia (when these groups were bound together prior to division and subsequent conflict) reported feeling more nostalgic for Yugoslavia and reported more contact with the ethnic groups that had resided in the former Yugoslavia (but not control ethnic groups).

National nostalgia is one type of collective nostalgia that is felt while self-categorizing as a citizen of a specific country, and is likely to be associated with particular intra- and intergroup attitudes and behavioral intentions. Just as personal nostalgia during times of change and upheaval can facilitate coping (e.g., attenuating loneliness) (Zhou et al., 2008), national nostalgia—a reverie for a country's good old days—may increase felt closeness to fellow natives during times of national stress or uncertainty. However, nostalgic revelry at the national level may exclude other citizens, such as recent immigrants or minorities (Smeekes and Jetten, 2019). Studies of national nostalgia among Dutch participants indicated that national nostalgia predicted prejudice toward religious minorities in the country (Smeekes et al., 2014) as well as prejudice toward Muslim countries (Smeekes, 2015). Notably, these outgroup attitudes were not predicted by personal nostalgia, which has been shown to be associated with decreased intergroup prejudice (Cheung et al., 2017). This distinction between personal and national nostalgia may lie in the extent to which outgroups pose an emotional threat to the self.

## National Nostalgia and Outgroup Threat

The intergroup threat theory (Stephan et al., 1999) posits that intergroup prejudice and hostility is largely explained by perceptions of threats to one's ingroup by an outgroup. In line with this theory, substantial evidence has found that intergroup prejudice is strongly influenced by both realistic and symbolic threat perception (Stephan et al., 2002; Mutz, 2018). Realistic threats are perceived threats to one's actual well-being, and typically include the domains of physical safety, political power, and economic security. Symbolic threats are more abstract, dealing with the cultural norms, ideologies, values, and traditions of one's ingroup (Stephan and Stephan, 2000). Realistic threats tend to be elicited from groups that are more economically powerful, whereas symbolic threats come about from marginalized outgroups who are perceived as highly dissimilar, and thus often inferior, to an ingroup (Stephan et al., 1999). Though these constructs are distinct and examined separately in the literature, there often is overlap between them, especially considering the demographic, economic, and social dynamics of some ingroups and outgroups. To be specific, when a marginalized minority grows in political, economic, or representative power, realistic and symbolic threats can be conflated (Craig and Richeson, 2014).

One salient factor in perceived threat for members of majority groups is the size of minority outgroups, with more threat being evoked by larger outgroups (Giles, 1977; Craig and Richeson, 2018) or even through messages endorsing diversity (Dover et al., 2016). In one notable set of studies by Craig and Richeson (2014), White American participants who read that the US population was becoming more diverse (relative to control conditions)—that the percentage of whites was dropping—reported more explicit (studies 1 and 3) and implicit (studies 2a and 2b) prejudice toward non-White outgroups and pro-White attitudinal bias. One possible explanation on why national and personal nostalgia are associated with different intergroup attitudes may be due to different levels of social categorization evoked, leading to differing levels of perceived threat. Personal nostalgia, which is associated with continuity of personal identity (Sedikides et al., 2015a) and evokes strong feelings of social connectedness, also has downstream implications for reducing anxiety and hostility toward outgroup members (for a review, see Sedikides and Wildschut, 2019). In contrast, feeling national nostalgia is associated with self-categorizing at the group level, evoking one's national identity (Smeekes and Verkuyten, 2015). Similar to how personal nostalgia may be evoked when feeling disconnection at the individual level, national nostalgia has been shown to be evoked in response to existential concerns about one's group-based identity, and may have the beneficial effect of reducing anxiety by bolstering perceptions of group continuity and connection (Smeekes et al., 2018). For example, trait national nostalgia among Dutch participants was positively associated with wanting to protect national ingroup identity (Smeekes, 2015). Similarly, a cross-national survey across 27 countries found that existential concerns about the future of one's country predicted increased collective nostalgia, which in turn predicted greater ingroup belonging and anti-immigrant sentiment (Smeekes et al., 2018). However, when the presence or power of outgroups is salient (e.g., chronically or by the rhetoric of politicians), national nostalgia may increase perceived threat. Moreover, ingroup continuity may be threatened by consideration of outgroups (Smeekes et al., 2018). This may be particularly true for people whose views of the national past are distorted—for example, when whites in the United States feel a longing for a (whiter and more homogenized) past that never was. Thus, national nostalgia could increase this fear of the future, leading to increased prejudice.

With the exception of a subsample of United States participants included in the cross-national study of Smeekes et al. (2018), this distinction has not been examined in the United States. Additionally, no studies have directly examined this theorized relationship in the context of political beliefs. Given that the tumultuous Trump years emphasized a number of political issues associated with national and ethnic identities, we extended this line of inquiry by examining whether perceived intergroup threat explains any found relationship between national nostalgia and endorsement of symbolic prejudice.

## National Nostalgia and Outgroup Perceptions in the Context of Political Messaging

Recent work has highlighted the prominence of national nostalgia in the rhetoric of right-wing populist political parties, and in particular its role in posing racial or national outgroups as scapegoats for perceived economic or cultural decline (Mols and Jetten, 2014; Smeekes et al., 2020). Political leaders often utilize national nostalgia in rhetorical strategy by emphasizing the discontinuity between a nation's past and present (Mols and Jetten, 2014), which then serves to evoke collective angst about group status (Smeekes et al., 2018). A content analysis of speeches by right-wing populist leaders in Western Europe found consistent themes of nostalgia for their country's “glorious past” while denigrating the country's present, as well as themes emphasizing that a) opponents of the party were the cause of this discontinuity between past and present, and b) increasing the country's strength and opposition to party opponents would return the nation to its former glory (Mols and Jetten, 2014). By emphasizing collective identity discontinuity, and then highlighting a potential scapegoat to blame for that discontinuity, populist leaders offer listeners an outlet for restoring psychological well-being by denigrating the outgroups believed to be responsible (Smeekes et al., 2018). Indeed, national nostalgia has been shown to explain support for right-wing populist policies and leaders via the denigration of immigrant and racial outgroups (Smeekes et al., 2020).

Similarly, the role of intergroup relations was a strong focus of Donald Trump's 2016 and 2020 presidential campaign rhetoric^1^. In the 2016 campaign, Trump borrowed Ronald Reagan's 1980 slogan, “Make America Great Again,” and emphasized claims that the United States had deteriorated from its former status. Along with these statements, he made numerous controversial statements on race, implying that changing demographics were, in part, to blame for this decline (Pettigrew, 2017). This led political pundits to claim that Trump's supporters were primarily White Americans who felt threatened by changing racial demographics and nostalgic for a past, whiter version of the United States. Exit polls from the 2016 presidential election appeared to support some of these claims, as White voters were the only racial demographic to support Donald Trump over Hillary Clinton, doing so by a large margin of 20 percentage points (CNN, 2016)^2^. Furthermore, several academic studies conducted in the wake of the 2016 election further supported the notion that intergroup attitudes played an important role in voters' choice to support Trump. Surveys conducted with representative panels found that support for Trump was most strongly predicted by negative attitudes toward the increased proportion of non-White US citizens in the population and anti-globalization attitudes (Hooghe and Dassonneville, 2018; Major et al., 2018; Mutz, 2018).

To build upon this research, the aim of our study was to directly examine how voters' propensity to feel national nostalgia may explain support for Trump's populist rhetoric as well as increases in racial prejudice in the United States following the 2016 presidential election (Edwards and Rushin, 2018). Furthermore, we hoped to highlight the unique role of perceived realistic and symbolic threats in shaping US voters' political attitudes. We thought it appropriate to examine both realistic and symbolic threats given the unique role of Black Americans in United States history and the ever-evolving racial and ethnic demographics of the United States, of which White Americans are becoming less of a majority (US Census Bureau, 2020).

## The Current Study

We examined the role of national nostalgia in propagating intergroup racial hostility above and beyond political orientation. We explored how national nostalgia relates to political and racial attitudes among voters who participated in the 2016 US presidential election. We also examined the interplay between national nostalgia, pro-Trump attitudes, outgroup prejudice, and perceived outgroup threat.

Although previous research examined survey data taken around the time of the 2016 presidential race (Hooghe and Dassonneville, 2018; Mutz, 2018), our data were collected ~1 year after the election, allowing us to see how our participants felt after President Trump had been in office for some time, and whether the nostalgic message of “Making America Great Again” still resonated with voters. Minimal work on national nostalgia has been conducted, and to date, nearly all of this work has been conducted outside of the United States; thus, this research would explore the potential link between national nostalgia and political attitudes as well as study the phenomenon in the US sociopolitical landscape. In addition, we included a validated measure of personal nostalgia in order to better examine the association between personal and national nostalgia as well as to assess whether each type of nostalgia might be associated with political attitudes.

## Hypotheses

We tested one specific hypothesis and three exploratory research questions, which were pre-registered on Open Science Framework (https://osf.io/mwh6n).

**Hypothesis 1**. National nostalgia would be positively related to pro-Trump attitudes (1a). No relationship was expected to be found between personal nostalgia and positive attitudes toward President Trump (1b).

**Research Question 1**. Will White or Republican identity be positively related to pro-Trump attitudes?

**Research Question 2**. Will national nostalgia be positively related to racial prejudice?

**Research Question 3**. Will the relationship between national nostalgia and racial prejudice be mediated by increased threat sensitivity?

## Method

### Participants

An *a priori* power analysis using G^*^Power (Faul et al., 2009) indicated a minimum of 132 individuals would be needed to detect a small correlation of *r* = 0.09^3^ with 95% power and α = 0.05. We recruited 252 US citizens who voted in the 2016 presidential election and identified as either White or Black (57.9% female, and 54.4% White). Participant age ranged from 18 to 79 (*M* = 36.34, *SD* = 12.68). Regarding political affiliation, 44.0% of the participants identified as Democrats, 25.4% Independent, 23.4% Republican, and 7.2% as Other. Participants were recruited through Amazon MTurk (www.mturk.com) during the Fall of 2017 and compensated $0.30 for completing the survey.

Regarding our sample demographics, White individuals comprised approximately 74% of the electorate in the 2016 election (Pew Research Center, 2018); however, we purposefully oversampled Black voters for the purposes of achieving appropriate statistical power for our analyses. Additionally, Republicans comprised ~31% of the electorate, with Democrats and Independents making up 35 and 34%, respectively. Thus, we feel that our sample is an accurate reflection of the 2016 US voters.

### Measures

#### Personal Nostalgia

The Southampton Nostalgia Scale (SNS; Routledge et al., 2008) measured personal nostalgia, operationalized as how frequently participants experience nostalgia and how significant participants felt nostalgic experiences were to them. The scale included seven items (e.g., “How valuable is nostalgia for you?”) rated from 1 (*Not at all*) to 7 (*Very much*). To build on past national nostalgia research (Smeekes et al., 2014), we use a validated measure of personal nostalgia (proneness to feeling personal nostalgia).

#### National Nostalgia

The National Nostalgia Scale (NNS; Smeekes et al., 2014, Study 1) measured participants' propensity to feel nostalgia on the basis of one's national ingroup membership. The scale included four items rated from 1 (*Very rarely*) to 5 (*Very frequently*) scale. The NNS used in this study was modified from the scale of Smeekes and Verkuyten (2015)^4^ to reflect American nationality [e.g., “How often do you long for the America (Netherlands) of the past?”].

#### Positive Attitudes Toward Trump

In terms of political attitudes, we wanted to assess positive sentiment toward the President as related to the experience of nostalgia. Therefore, we used a modified version of the State Functions of Nostalgia Scale **(**SFN; Hepper et al., 2012), which measures the extent to which nostalgia confers the positive benefits of social connectedness, well-being, self-regard, and overall positive affect. Each item was modified to assess how participants experienced these benefits as they related to Donald Trump's presidency. This scale consisted of 16 items (e.g., “Thinking about the election of Donald Trump makes me feel protected/happy/life is worth living”), that were rated on a 1 (*Not at all*) to 5 (*Extremely*) scale.

#### Outgroup Threat Perception

The Realistic Threat Scale (RTS; Stephan et al., 2002) was employed to measure realistic threat perceptions (e.g., of social or economic harm) of Black individuals. The scale was examined only among White participants. The measure includes 12 items (e.g., “African Americans hold too many positions of power and responsibility in this country”) rated on a 1 (*Strongly disagree*) to 7 (*Strongly agree)* scale.

#### Racial Prejudice

The Symbolic Racism Scale (SRS; Henry and Sears, 2002) was used to assess cognitive and affective dimensions of racial prejudice toward Black individuals. The measure consisted of eight items (e.g., “It's really a matter of some people not trying hard enough; if Blacks would only try harder they could be just as well off as Whites.”) rated on a 1 (*Strongly disagree*) to 4 (*Strongly agree*) scale.

#### Political Measures

Participants reported their political orientation on a scale ranging from 1 (Very Liberal) to 7 (Very Conservative). Participants also chose which political party they most strongly identified with (Democrat, Republican, Independent, or Other). Participants then indicated which political candidate they voted for in the 2016 presidential election (Hillary Clinton, Donald Trump, or Other). They then responded to the question “How much do you feel like we need to ‘Make America Great Again’?” on a 1 (*Not at all*) to 7 (*Extremely*) scale. Finally, participants reported their country of origin and whether English was their native language.

#### Ethnic Identity Salience

The Multi-Ethnic Identity Measure—Revised (MEIM-R; Phinney and Ong, 2007) was used to determine the centrality of participants' racial/ethnic backgrounds to their sense of self. The scale contains such as “I have a strong sense of belonging to my ethnic group,” and each item was rated on a scale of 1 (*Strongly disagree*) to 5 (*Strongly agree*) scale.

#### Demographics

Participants last reported their gender, age, and racial identity.

### Procedure

Participants signed up through Amazon Mturk to complete an online survey about their attitudes toward the past, race, and politics. After indicating their informed consent, participants responded to all study measures and items in the order described above. All responses were collected over a single, 1 week period in the Fall of 2017 to avoid history artifacts in the data. Additionally, all participants passed attention checks ensuring that they were properly attending to questionnaire items. For the purposes of this survey, missing more than two attention check items indicated insufficient attention and warranted non-inclusion of that participant's data.

## Results

Descriptive statistics and zero-order correlations are displayed in Table 1. To test our hypotheses, we conducted a series of hierarchical linear regression models and bootstrapped mediation and moderation analyses to assess the relationship between nostalgia (national and personal) and political and intergroup attitudes using SPSS v. 20 and Hayes' PROCESS macro v.3 (Hayes, 2013). Following these baseline models, we also support our findings using path analyses employing maximum likelihood estimation using IBM AMOS v. 26 (Due to a computer error, the national nostalgia data from 72 participants were unusable, reducing the *n* for analyses including national nostalgia to 193, still above the target based on the power analysis).

**Table 1:** Descriptive statistics and bivariate correlations among study variables.

**Variable**	**1**	**2**	**3**	**4**	**5**	**6**	**7**	**8**	**9**	**10**	**12**	**13**	**14**	**M/Percent**	**SD**
1	Ethnic/Racial Identity Salience	0.91													3.38	0.92
2	Personal Nostalgia	0.15^**^	0.92												4.85	1.19
3	National Nostalgia	0.18^**^	0.32^***^	0.90											2.85	1.16
4	Pro-Trump Attitudes	0.24^***^	0.08	0.49^***^	0.97										2.62	1.41
5	Outgroup Threat Perception	0.07	−0.01	0.44^***^	0.62^***^	0.98									2.38	1.52
6	Racial Prejudice	0.08	0.07	0.47^***^	0.63^***^	0.63^***^	0.84								0.34	0.23
7	MAGA	0.14^**^	0.02	0.52^***^	0.61^***^	0.54^***^	0.65^***^	–							3.33	2.72
8	Political Orientation	0.12	0.01	0.46^***^	0.59^***^	0.47^***^	0.66^***^	0.67^***^	–						3.48	1.76
9	Republican	0.08	0.01	0.33^***^	0.52^***^	0.35^***^	0.51^***^	0.60^***^	0.63^***^	–					23.4%	–
10	Democrat	0.08	0.00	−0.28^***^	−0.35^***^	−0.25^***^	−0.38^***^	−0.47^**^	−0.53^***^	−0.49^***^	–				44.0%	–
11	Independent	−0.15^*^	−0.03	0.05	−0.14^*^	−0.05	−0.05	−0.02	0.02	−0.32^***^	−0.52^***^	–			25.4%	–
12	Gender	−0.05	−0.13^*^	−0.07	0.18^**^	0.18^**^	0.19^**^	0.10	0.15^*^	0.05	−0.12	0.10	–		57.1% (F)	–
13	Age	0.01	0.10	0.08	−0.04	−0.20^**^	−0.08	0.02	0.01	−0.03	0.03	0.03	−0.03	–	36.34	12.68
14	Race	0.33^***^	−0.08	−0.12	−0.04	−0.07	−0.17^**^	−0.09	−0.07	−0.04	0.20^**^	−0.17^***^	−0.12	−0.17^**^	54.4% (EA)	–

* *p < 0.05*,

** *p < 0.01*,

*** *p < 0.001 (two-tailed)*.

*Gender coded with female = 0, male = 1. Race coded with 0 = European American, 1 = African-American. MAGA = “How much do you believe we need to 'Make America Great Again'?” For analyses including National Nostalgia, N = 193; for all other correlations, N = 252. Cronbach alphas are displayed on the diagonal*.

### Main Hypothesis

We first assessed whether national nostalgia and personal nostalgia would be related to pro-Trump attitudes in the ways previously predicted. National nostalgia and personal nostalgia proneness were entered simultaneously in step 2 of the model to identify their unique relationship with attitudes toward Trump. In step 1 of the hierarchical model, political orientation significantly predicted pro-Trump attitudes such that higher conservatism was associated with more positive attitudes of Trump, β = 0.59 *t*(192) = 10.08, *p* < 0.001. In step 2 of the model, national nostalgia was associated with more pro-Trump attitudes above and beyond political affiliation, β = 0.30, *t*(192) = 4.43, *p* < 0.001, supporting Hypothesis 1a. In contrast, personal nostalgia was not associated with pro-Trump attitudes above and beyond political orientation, β = −0.07, *t*(192) = −1.13, *p* = 0.259. Nostalgia predicted a significant proportion of variance in attitudes above and beyond political orientation, *F*_(2, 189)_ = 9.90, *p* < 0.001, R^2^Δ = 0.06.

**Figure 1:**
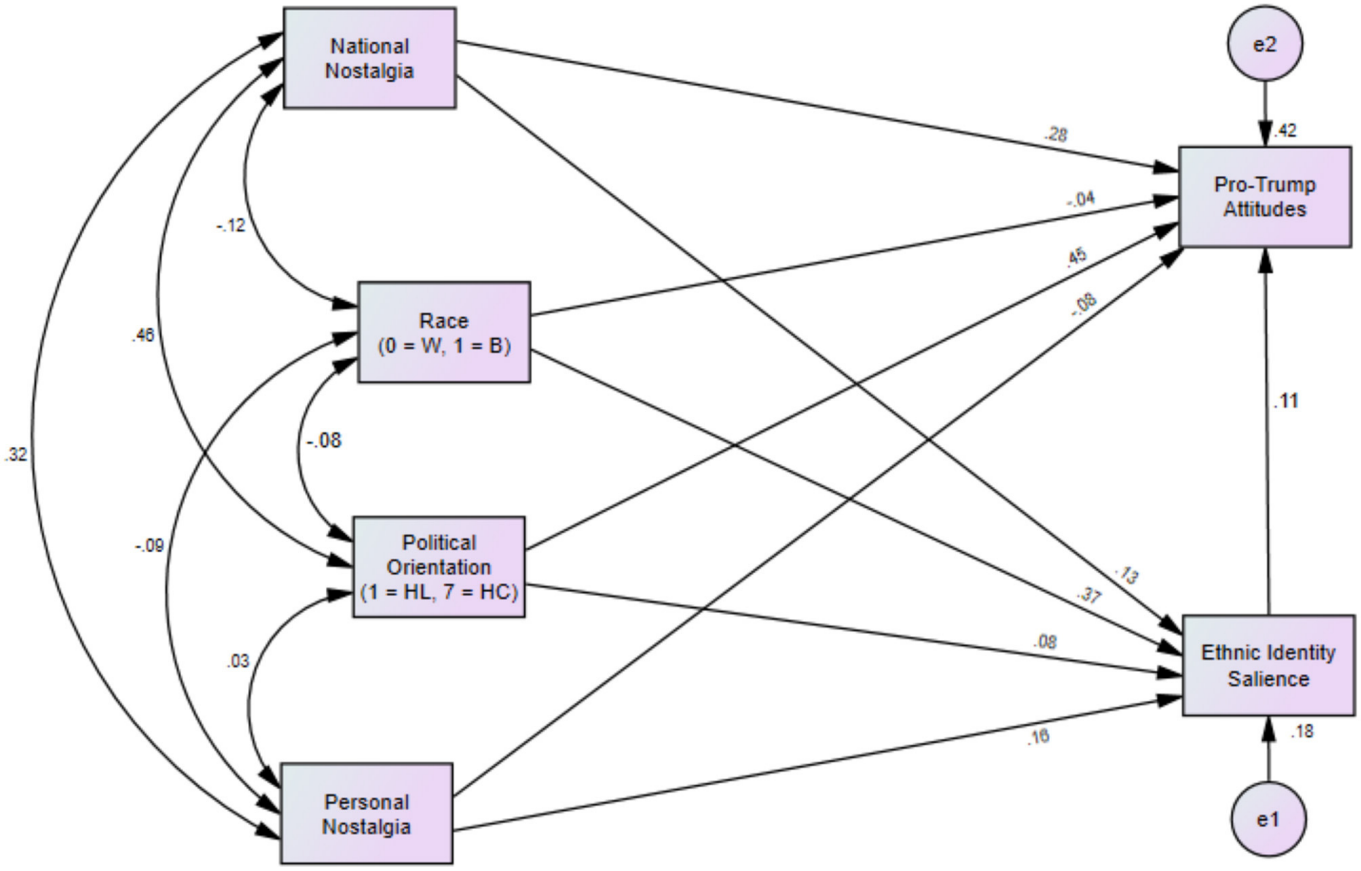
Path analysis of relationships between national/personal nostalgia, ethnic identity, and pro-Trump attitudes (Model 1). Note. Path coefficients represent standardized estimates.

To examine this relationship in a consolidated path model^5^, Figure 1 displays Path Model 1, quantifying the relationship between national and personal nostalgia and race, political orientation, ethnic identity salience, and pro-Trump attitudes. The model fit the data somewhat weakly due to the lower sample size [χ2(1) = 23.01, *p* < 0.001; CFI = 0.89; RMSEA = 0.34; SRMR = 0.03]. As shown in Model 1, Hypothesis 1 was again supported: national nostalgia predicted pro-Trump attitudes (β = 0.24, *p* < 0.001), whereas personal nostalgia was unrelated to pro-Trump attitudes (β = −0.08, *p* = 0.156).

### Research Question 1

**Figure 2:**
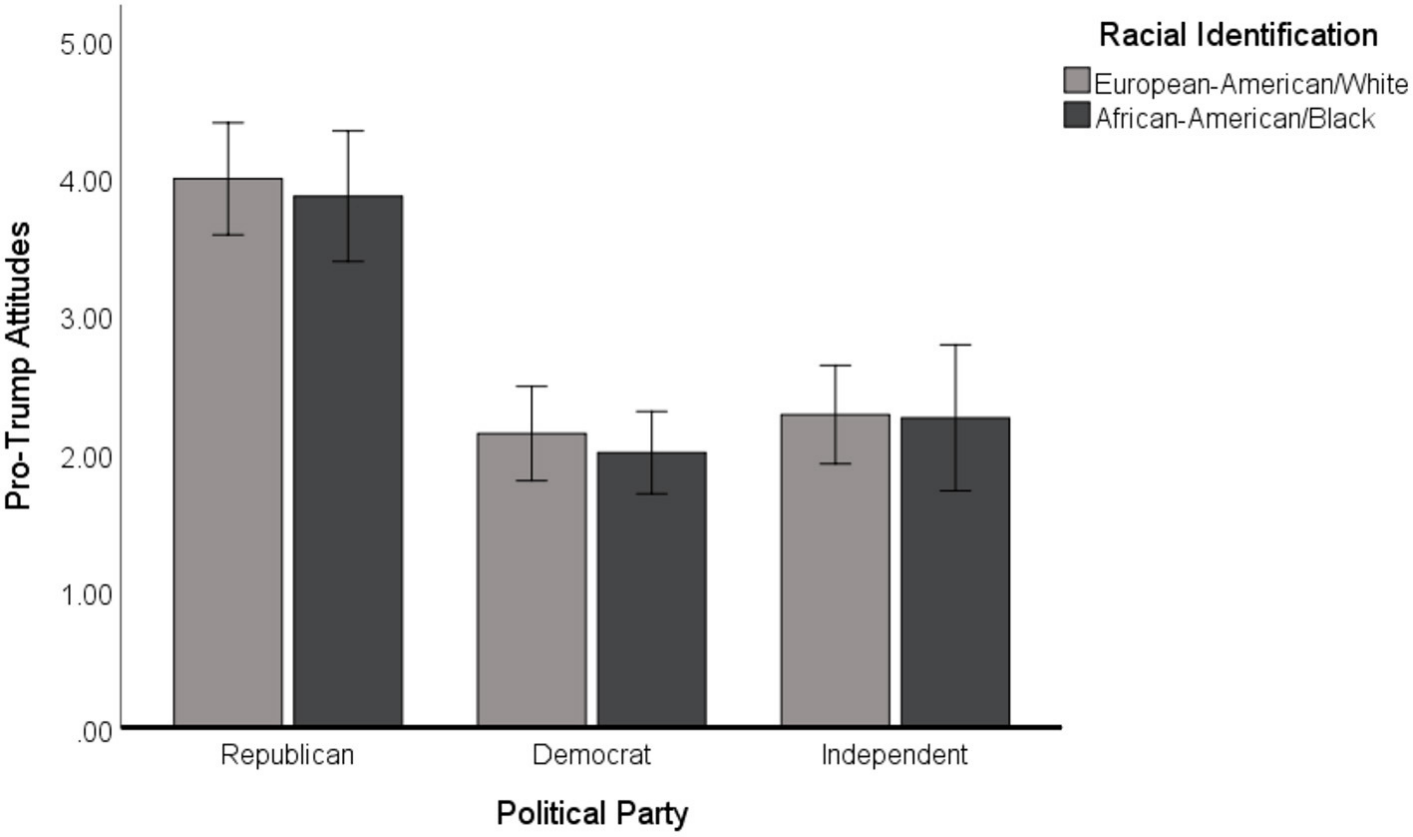
Relationship between political party affiliation and pro-Trump attitudes by racial identity. Note. Error bars represent 95% CIs around the mean for each subgroup.

To assess whether there was an association between race, political affiliation, and pro-Trump attitudes, we ran a 2 (Racial Identification) × 3 (Political Party Affiliation) ANOVA. Racial identification was coded with 0 = White/European-American, 1 = Black/African-American (shortened to W/EA and B/AA going forward). Political party affiliation was coded as 1 = Republican, 2 = Democrat, and 3 = Independent and were analyzed using an indicator multicategorical contrast. For the purposes of this analysis, data from participants who did not identify with one of these three major political groups were excluded. The model included 59 Republicans (34 W/EA, 25 B/AA), 111 Democrats (48 W/EA, 63 B/AA), and 64 Independents (44 W/EA, 24 B/AA). The factorial model found that political party affiliation was the only significant predictor of holding positive attitudes toward President Trump, *F*_(2, 228)_ = 47.73, *p* < 0.001, partial η^2^ = 0.30, with Republicans (*M* = 3.94, *SD* = 1.22) more in favor of the president than their Democratic (*M* = 2.06, *SD* = 1.26) or Independent (*M* = 2.27, *SD* = 1.06) counterparts. There was no main effect of participant race (Black or White) on attitudes toward the President, *F*_(1, 228)_ = 0.47, *p* = 0.57, nor was there an interaction between political party affiliation and participant race, *F*_(2, 228)_ = 0.05, *p* = 0.96. Figure 2 displays these results.

**Figure 3:**
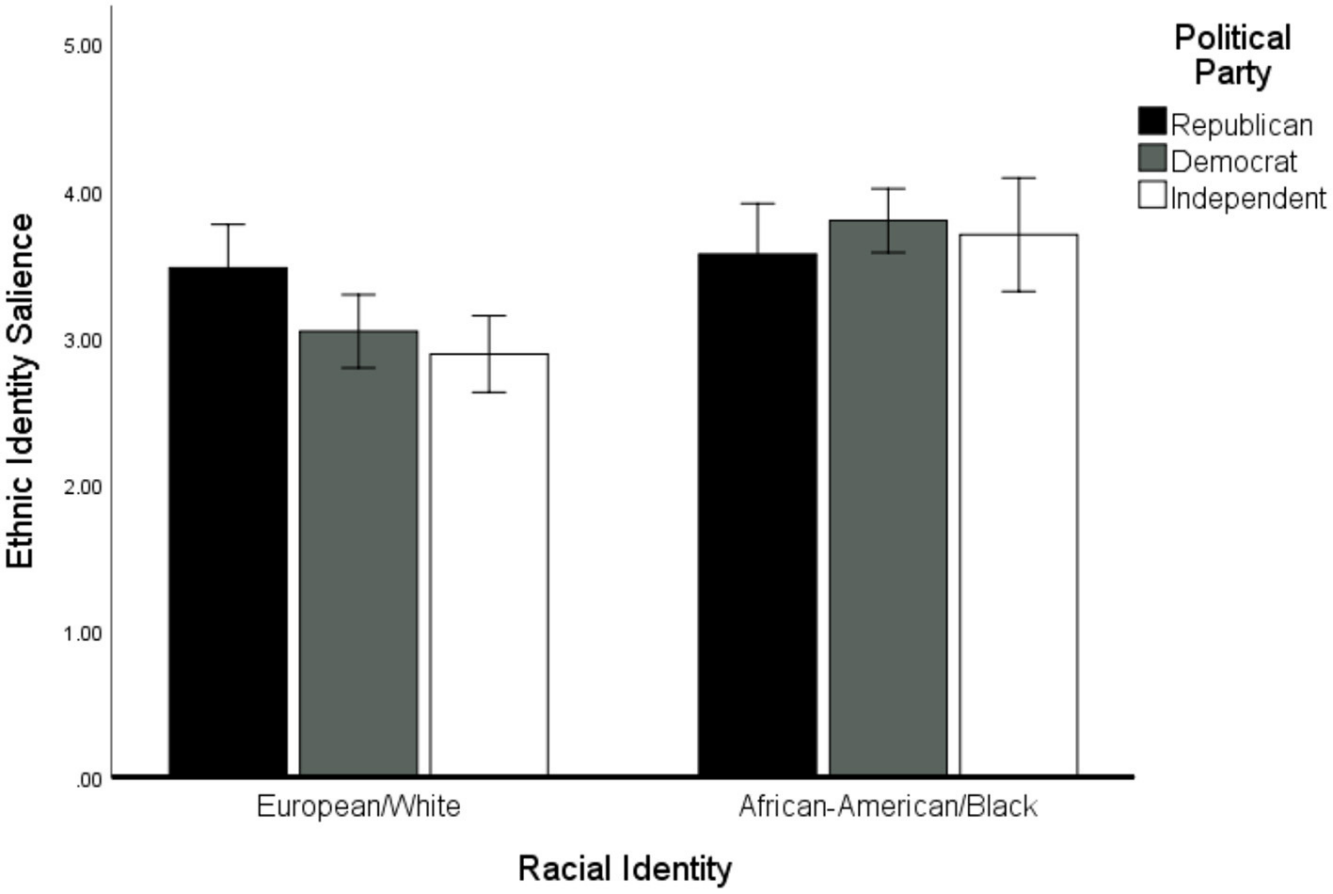
Racial identity salience among Black/African-American and White/European-American participants of different political affiliations (Republican, Democrat, Independent). Note. Error bars represent 95% CIs around the mean for each subgroup.

To explore these results further, we examined whether ethnic identity *salience*, rather than race itself, may be an important qualifying variable in explaining pro-Trump attitudes. We examined whether political party (dummy coded with Republican = 0 to compare against Democrats and Independents) interacted with race (dummy coded with W/EA = 0) to predict racial identity salience (measured by the MEIM) using Hayes' PROCESS macro v. 3.4 (model 1). We conducted a bootstrapped moderation analysis with 5,000 resamples, which indicated a significant higher-order interaction effect between political affiliation and race to predict ethnic identity salience, *F*_(2, 228)_ = 3.23, *p* = 0.041, R^2^Δ = 0.024. An analysis of the simple slope effects indicated that there was a stronger difference in ethnic identity salience among White participants compared with Black participants. White Republicans (*M* = 3.47, *SD* = 0.92) reported that their racial identity was significantly more important to them than their White Democratic [*M* = 3.04, *SD* = 0.91, *b* = −0.43, 95% CI = (−0.82, −0.04)] and Independent counterparts [*M* = 2.89, *SD* = 0.92, *b* = −0.59, 95% CI = (−0.98, −0.19)]; simple slope difference *F*_(2, 228)_ = 4.49, *p* < 0.001. In contrast, no significant difference in racial identity salience was found among Black/African-American participants; simple slope difference *F*_(2, 228)_ = 0.63, *p* = 0.537. In fact, an analysis of the simple main effect of race among Republicans indicated that White Republicans felt their racial identity was equally as important to them as Black participants; *M* = 3.73, *SD* = 0.83, *b* = 0.24, 95% CI = (−0.16, 0.63). Black Democrats [*b* = 0.60, 95% CI = (0.37, 0.83)] and Black Independents (*b* = 0.97, 95% CI = (0.57, 1.36)] reported significantly higher ethnic identity salience compared with White Democrats and Independents (see Figure 3).

We also examined whether racial identity salience qualified the relationship between national nostalgia and pro-Trump attitudes. A moderation analysis using Hayes' PROCESS macro (model 1) indicated that higher racial identity salience somewhat strengthened the relationship between national nostalgia and positive attitudes toward Trump, but only among White participants; Δ*R*^2^ = 0.03, *F*_(1, 77)_ = 3.94, *p* = 0.051. Among those low in racial identity salience, national nostalgia was unrelated to attitudes toward Trump; *b* = 0.27, 95% CI = (−0.03, 0.58). Those moderate [*b* = 0.43, 95% CI = (0.18, 70)] and high [*b* = 0.64, 95% CI = (0.31, 0.97)] in racial identity salience showed a strong relationship between national nostalgia and pro-Trump attitudes.

**Figure 4:**
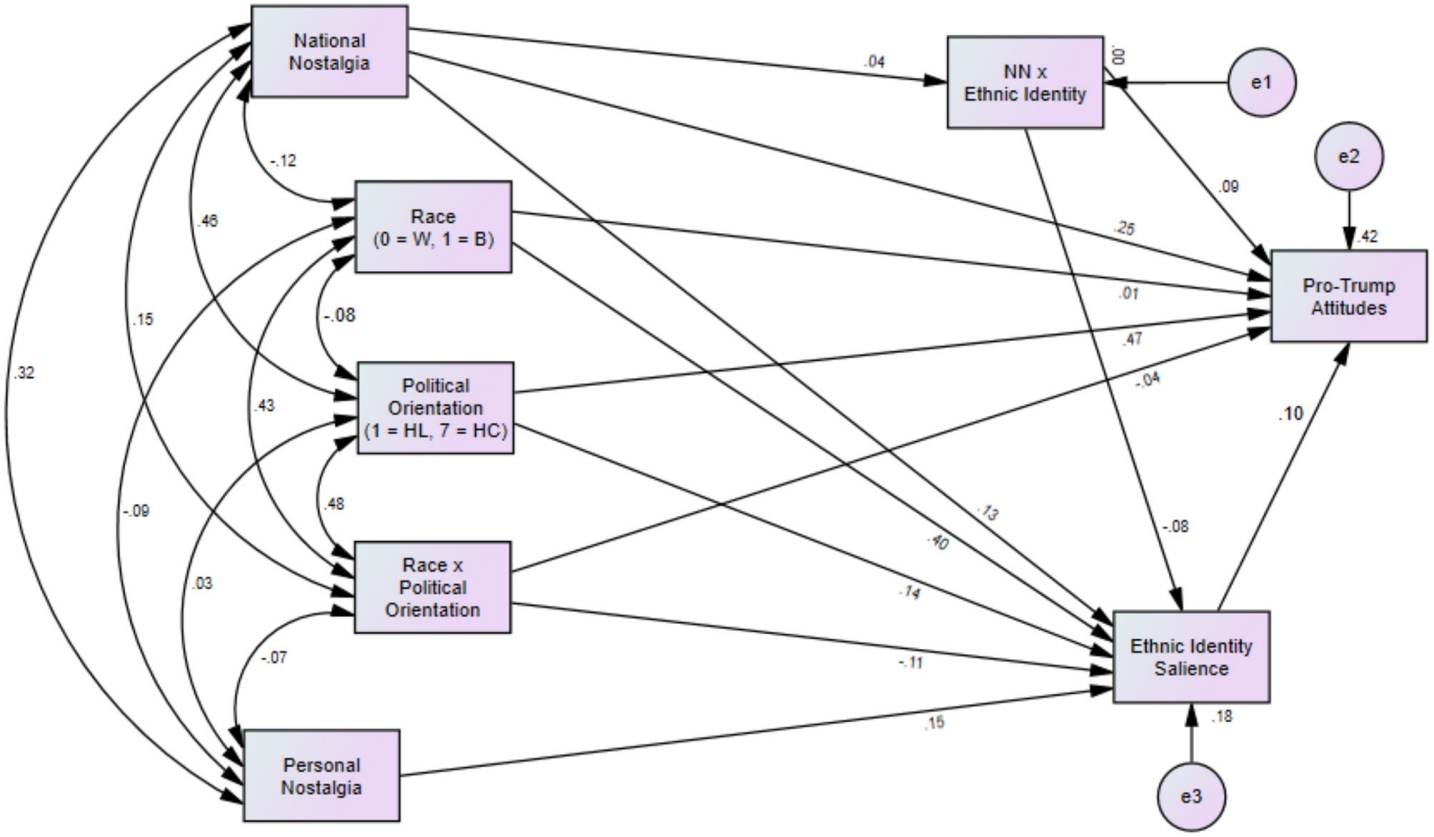
Path analysis estimating interaction effects (race × political orientation and ethnic identity × nostalgia) on pro-Trump attitudes. Note. Path coefficients represent standardized estimates.

As a final examination of Research Question 1, a second path model (Path Model 2, Figure 4) was compared with Path Model 1 to again examine the interaction between nostalgia and ethnic identity (on pro-Trump attitudes), and the interaction between political orientation and race (assessing its relationship with ethnic identity). When interpreting this model, it is important to note that path models are generally considered ineffective in examining interaction effects (Meyers et al., 2016). Path Model 2 showed much improved fit relative to Path Model 1 [χ2(10) = 40.47, *p* < 0.001, CFI = 0.95, RMSEA = 0.09^6^; SRMR = 0.05]. Likely due to the limitations of path models to compute interaction effects, in contrast to what was shown in the PROCESS model, the interaction between race and political orientation (measured on a continuous scale) was not significantly associated with ethnic identity (β = −0.08, *p* = 0.210). Additionally, the interaction term between national nostalgia and ethnic identity was no longer associated with pro-Trump attitudes (β = 0.13, *p* = 0.607). This suggests that for White participants, greater national nostalgia was associated with increased ethnic identity.

### Research Question 2

**Figure 5:**
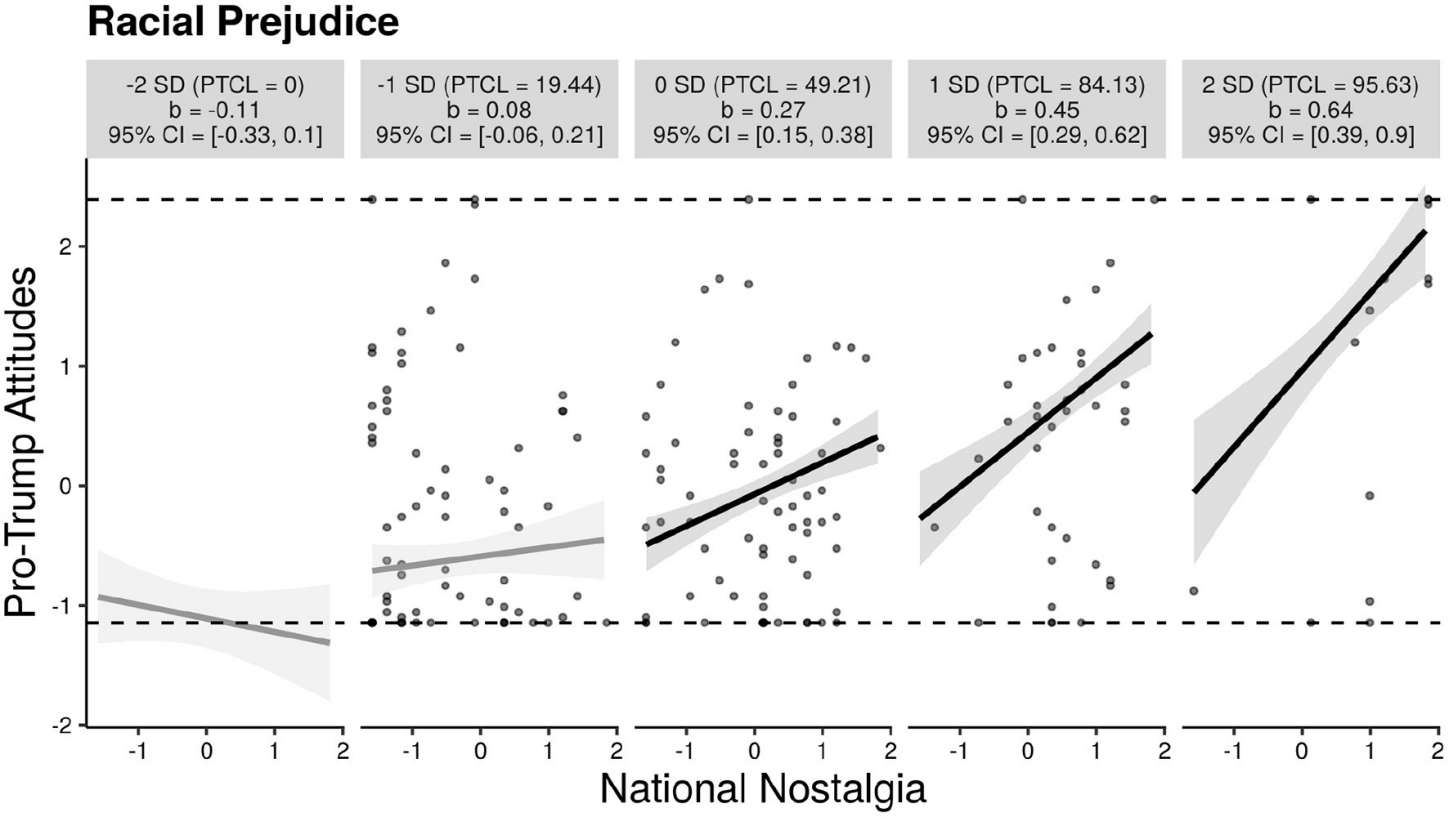
Relationship between national nostalgia and pro-Trump attitudes moderated by anti-Black racial prejudice. Note. Plots display simple slopes at −2, −1, 0, +1, and +2 SDs away from the mean of racial prejudice for all participants. PTCL, percentile.

We next examined whether national nostalgia was positively related to racial prejudice. Bivariate correlations indicated that national nostalgia was positively associated with both anti-Black racial prejudice measured by the Symbolic Racism Scale (SRS) as well as perceived realistic threat measured by the Realistic Threat Scale (RTS, see Table 1). To further examine the link between national nostalgia and racial prejudice, we tested whether racial prejudice moderated the link between national nostalgia and positive attitudes toward President Trump using Hayes' PROCESS macro (model 1) with 5,000 resamples. A significant moderation effect was identified. Participants reporting higher prejudice exhibited a stronger relationship between national nostalgia and pro-Trump attitudes; Δ*R*^2^ = 0.05, *F*_(1, 178)_ = 19.60, *p* < 0.001. Simple slopes were calculated and visualized using the interActive online utility, and are presented in Figure 5 (McCabe et al., 2018). The relationship between national nostalgia and positive attitudes toward Trump was non-significant at low levels of prejudice (those at least −1 SD below the mean of SNS). However, for those moderate to high in racial prejudice (0, +1, or +2 SDs above the mean of SNS), national nostalgia positively predicted pro-Trump attitudes (see Figure 5). Interestingly, this effect was found separately for both White [Δ*R*^2^ = 0.03, *F*_(1, 77)_ = 5.93, *p* = 0.02] and Black participants [Δ*R*^2^ = 0.09, *F*_(1, 97)_ = 17.44, *p* < 0.001], but there was no significant three-way interaction between national nostalgia, prejudice, and race (*p* = 0.14), so the results in Figure 5 are displayed for all participants.

### Research Question 3

Will the relationship between national nostalgia and racial prejudice be mediated by increased threat sensitivity?

**Figure 6:**
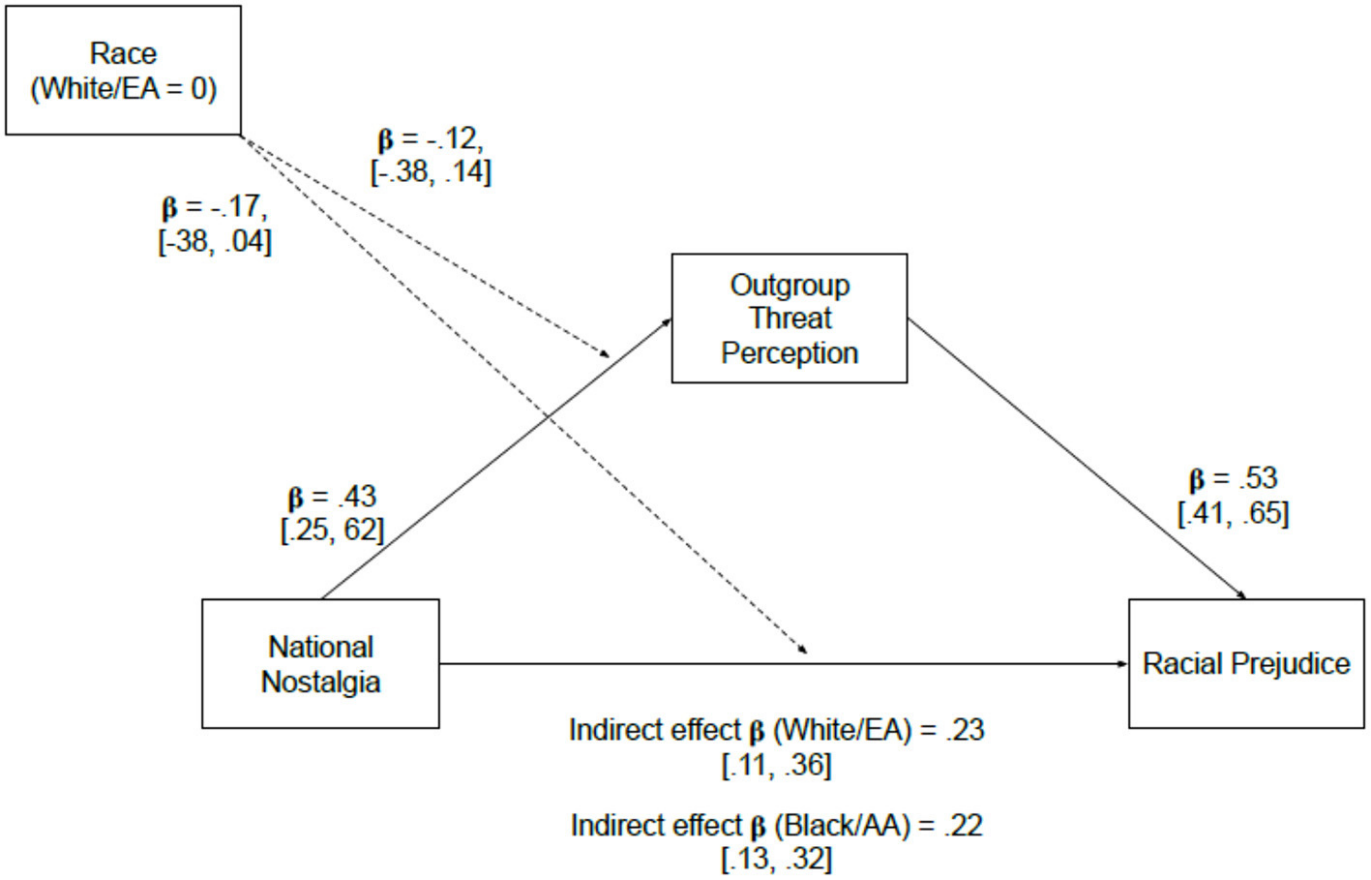
Mediation of national nostalgia relationship with racial prejudice by outgroup threat perception, moderated by participant race.

We last examined whether the relationship between national nostalgia and racial prejudice would be mediated by outgroup threat perception (measured by the Realistic Threat Scale, RTS). A moderated mediation model was constructed using Hayes' PROCESS macro (model 8) to assess whether the proposed mediational effect might differ between European-American and African-American participants. As shown in Figure 6, the model indicated a significant indirect effect of national nostalgia on prejudice through the mediator of perceived threat for both White/EA participants [β = 0.23, 95% CI = (0.12, 0.36)] and Black/AA participants [β = 0.22, 95% CI = (0.13, 0.32)]. The mediational indirect effect did not differ by participant race; β = 0.07, 95% CI = (−0.15, 0.13).

**Figure 7:**
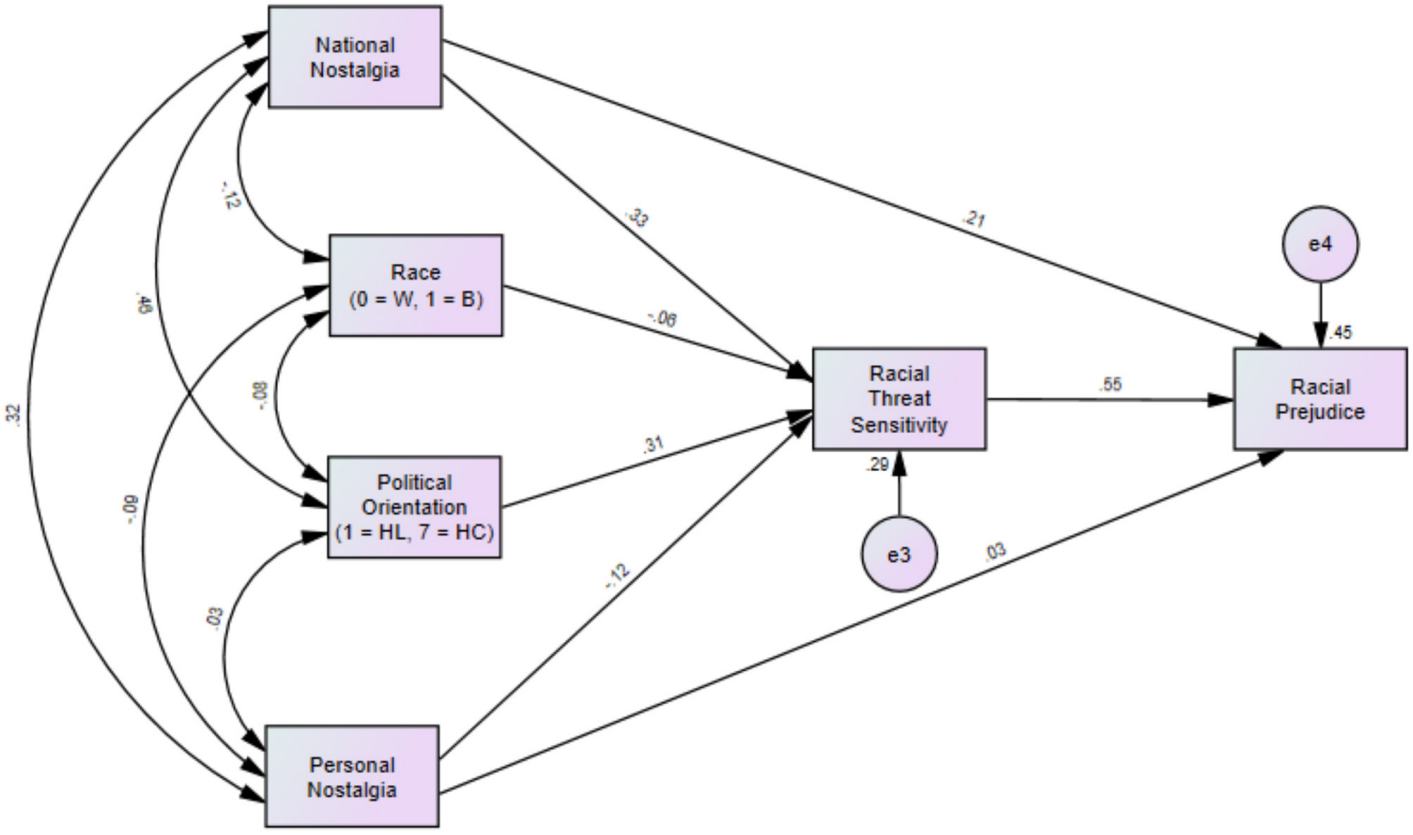
Path analysis of relationships between national/personal nostalgia and prejudice, mediated by racial threat sensitivity (Model 3). Note. Path coefficients represent standardized estimates. Indirect effect of national nostalgia on racial prejudice through racial threat sensitivity was significant [β = 0.18; 95% bias-corrected CI (0.10, 0.26)].

To examine this question in the context of a path model, Path Model 3 (Figure 7) displays the proposed relationships between national nostalgia and racial prejudice. Model 3 showed a moderate fit with the data, χ(2) = 65.80, *p* < 0.001; CFI = 0.79; RMSEA = 0.41; SRMR = 0.07). When accounting for political orientation, race, national nostalgia, personal nostalgia, racial threat sensitivity, and racial prejudice in a structural equation mediation model, national nostalgia directly predicted racial prejudice (β = 0.21, *p* < 0.001), whereas personal nostalgia did not (β = 0.03, *p* = 0.581). The relationship between national nostalgia and racial prejudice was significantly mediated by threat sensitivity [indirect effect β = 0.18, 95% bias-corrected CI (0.10, 0.26)]. Interestingly, personal nostalgia also showed a weak indirect effect on national nostalgia via threat sensitivity, but in a negative direction [indirect effect β = −0.07, 95% bias-corrected CI (−0.14, −0.01)]. This suggests that greater personal nostalgia may weakly predict lower racial prejudice via reduced racial threat sensitivity.

## Discussion

In our study, national nostalgia was associated with more positive feelings about President Trump, as well as increased perceived racial threat among White respondents. In contrast, personal nostalgia was unrelated to support for Trump or perceived racial threat. When assessed in a path model, personal nostalgia was actually associated indirectly with lower anti-Black prejudice via decreased racial threat sensitivity. These findings align with evidence from samples outside the United States (e.g., Smeekes and Verkuyten, 2015; Smeekes et al., 2020) that personal and national nostalgia are distinct experiences with unique ramifications for intergroup attitudes and relations. Though our overall finding that national nostalgia predicted Trump support could reflect a strong semantic connection between Trump and its 2016 presidential campaign slogan, it also may point to the appeal of Trump's campaign—and its right wing, populist sentiments—among those initially prone to feeling national nostalgia. To better answer this question, our next analyses investigated more closely the relationship between national nostalgia and identity.

Our first research question asked whether identity was associated with national nostalgia. We found partial evidence for this idea, as Republican participants expressed greater positive attitudes toward Trump. However, there was no evidence of a relationship between race and support for the President. At first glance, this finding does not align with media narratives and political polling suggesting that Trump's messaging appealed mostly to White voters. However, although race itself did not predict support for the President, racial *identity salience* moderated the link between national nostalgia and pro-Trump attitudes. White Republicans felt more strongly connected to their racial identity than Whites who identified as either Democrats or Independents. White Republicans also expressed significantly more positive feelings toward the President than other groups. In fact, they rated their racial identity as important as Black participants in our sample. This is notable, as it evidences further support for the influence of White identity on political attitudes (Schildkraut, 2015). As members of the majority group, White individuals typically are less likely to think of themselves in terms of race than people of color, for whom race is a more centralized component of their identity (Steck et al., 2003).

This finding suggests that the perception of demographic changes and threats to the dominant ingroup in the United States may indeed have been a critical factor in voters' choice to support Trump. Some research suggests that, in the current political climate, White Americans may increasingly identify with their Whiteness, as a result of threat resulting from shifting racial demographics (Jardina, 2019). However, there is an issue of causality, as these correlational data could indicate that the perception of such a threat may increase the salience of one's racial identity. This threat may be perceived more strongly by those for whom a White racial identity was already a more central part of their self-concept. For instance, Schildkraut (2015) found that White Americans with higher White identity scores, along with heightened perception of discrimination against Whites and feeling a sense of linked fate with other White Americans, were substantially more likely to politically endorse a White candidate. This suggests that the threat to White identity, along with other related constructs, may influence political attitudes and may also offer an explanation on why leaders invoking national nostalgia may be so attractive to some individuals. This type of rhetoric typically emphasizes collective identity discontinuity in order to foment anxiety about the state of the country while simultaneously offering a restorative outlet by identifying racial outgroups as scapegoats.

The role of intergroup attitudes was apparent when examining the relationship between national nostalgia and pro-Trump support. We found that national nostalgia significantly predicted racial prejudice and that this relationship was mediated by perceived outgroup threat. Interestingly, this mediational effect was found among both White/EA and Black/AA participants, although the lack of a significant interaction effect may have been due to lower power. Additionally, we found a stronger relationship between national nostalgia and pro-Trump attitudes among those who reported more prejudice toward Black individuals. These findings align with evidence that group emotions motivate intergroup attitudes and, in particular, outgroup derogation when outgroups are perceived to be a threat (Smith et al., 2007; Wildschut et al., 2014). In particular, these findings align with converging evidence that the content of collective nostalgia—what individuals perceive to be “the good old days” for their identity group—reflects salient sources of perceived threat (Wohl et al., 2020). This conceptual model, highlighting the content of collective nostalgia, also explains differences between the emotional outcomes of personal and national nostalgia. Whereas, personal nostalgia enhances feelings of belonging by evoking memories of positive intrapersonal experiences in the face of ostracism or loneliness, national nostalgia may enhance belongingness by evoking positive thoughts about the “good old days” when one's group was perceived to be higher in status or less threatened by outgroups. It is also possible that national nostalgia, like personal nostalgia, may enhance feelings of continuity in its own way, by allowing individuals to feel connected to a time in which they believed their ingroup identity was less threatened or somehow stronger. Recent work supports the notion that, analogous to personal nostalgia, enhancing feelings of self-continuity (Sedikides and Wildschut, 2019), national nostalgia is linked to feelings of ingroup continuity (Smeekes et al., 2018). A study across 27 countries found that national nostalgia was associated with stronger feelings of ingroup continuity (Smeekes et al., 2018); ingroup belonging but not prejudice (outgroup rejection) appeared to mediate this link. Since relatively little research on collective nostalgia, particularly national nostalgia, has been undertaken, future work should examine these questions via multiple methods, particularly longitudinal and experimental designs, which can identify whether and to what extent self-continuity is enhanced by (or itself predicts) collective nostalgia in response to outgroup threat.

### Constraint on Generalizability

These data were obtained from a cross-sectional group of US Mturk workers in the Fall of 2017, so these results are most generalizable to American middle-aged populations (Huff and Tingley, 2015). Additionally, these considerations of intergroup threat perception and prejudice are most generalizable to White/EA and Black/AA social groups within the United States, and future analysis of national nostalgia should continue to assess different ethnicities, races, and other relevant social categories.

### Future Directions

These findings raise the question on whether national nostalgia stems from a desire by some to go back in time, due to perceived group identity threats. Future research should employ longitudinal or experimental methods, such as manipulating identity threat, to examine whether national nostalgia arises as a defense against perceived threats to one's ingroup. Relatedly, it is only recently that national nostalgia has been manipulated (Smeekes and Verkuyten, 2015; Wohl et al., 2020), as the majority of national nostalgia research has been at the trait level. Further work evoking national nostalgia in experimental contexts would allow us to better understand how this emotion interacts with intergroup attitudes, prejudice, and feelings of threat. We should also continue to examine how the importance of racial identity, including white racial identity, plays a role in their political attitudes and actual voting behavior. The need for further research in this area has grown substantially in recent years, especially in light of events such as those that took place in Charlottesville in 2017 and at the US Capitol Building in early 2021, in which large groups of White Nationalists gathered in events that ultimately turned violent.

An additional question to be explored is the extent to which national nostalgia operates within specific cultures and nations. Although Trump's presidential tenure has ended, the importance of these findings is not constrained only to the rhetoric from his campaign. Rather, the use of national nostalgia in political communication is widespread (Mols and Jetten, 2014; Smeekes et al., 2020) and has far-reaching implications. Future research should examine the role of national nostalgia in shaping attitudes toward demagogues in a variety of settings and when considering a variety of societal outcomes. Our findings suggest that national nostalgia may influence intergroup attitudes as a group-based emotion broadly through evoking positive emotions about one's national group identity. However, the nature of the construct suggests it may also operate through evoking shared historical knowledge and schemas about one's group within a specific nation. The phrase “make America great again” and other nostalgic political rhetoric is particularly controversial in the US because minority groups have achieved significant advances in civil rights in recent history, and a call to return to a former time may imply a call for a return to a former and less egalitarian social hierarchy. Future research on national nostalgia should explore the nuances of this emotion and its expression among various ethnic and social groups in different countries. Expressions of national nostalgia may evoke intergroup hostility to a lesser extent within nations with different histories.

Future research might also examine the extent to which perceptions of outgroup threat stem from realistic (e.g., economic) vs. symbolic (e.g., social/moral) concerns. Prior research has theorized that symbolic threats (rather than realistic threats) may be more psychologically influential on voter support for right-wing populist ideology, as concerns about immigration and intergroup relations tend to emphasize the importance of preserving cultural homogeneity (Smeekes et al., 2020). Understanding the source and salience of perceived economic and cultural threats could help inform interventions to assuage anxiety, thus reducing prejudice toward outgroups. Finally, with the ever-evolving demographic makeup of the United States (as well as many other countries), further work in this area should include individuals who identify with other racial groups beyond White or Black, and should also be expanded to look at different identities such as gender, sexual orientation, religion, immigrant status, social class, education level, and nation of origin.

### Coda

National nostalgia, a form of collective nostalgic experience, is a promising lens through which to analyze attitudes, such as political and prejudicial attitudes, particularly when combined with assessments of identity salience and perceived outgroup threat. Research to date on national nostalgia is relatively new. Although this phenomenon has been studied elsewhere (mostly in European and Asian nations), this is the first study, to our knowledge, to examine the US political landscape. Personal nostalgia—a wistful longing for one's personal past—does not have the same associations with political and group attitudes, and only moderately correlates with national nostalgia. In contrast, national nostalgia, particularly in combination with white identity salience and outgroup threat perception, predicted both prejudice and political attitudes.

There may be some irony in the possibility that national nostalgia may include beliefs for a past that never was; in this case, an America that was not as white as some recollect. Nevertheless, these national nostalgic feelings appear to be linked to important social attitudes, and thus are worthy of further investigation.

## Data Availability Statement

The datasets presented in this study can be found in online repositories. All reported study hypotheses, measures, and methods were preregistered through the Open Science Framework, available at https://osf.io/mwh6n. De-identified data and study information can be viewed at https://osf.io/6j4gm/. Some survey measures listed in the preregistration were not analyzed in this study and therefore not listed in this report.

## Ethics Statement

The studies involving human participants were reviewed and approved by Virginia Commonwealth University IRB. The patients/participants provided their written informed consent to participate in this study.

## Author Contributions

AB, AC, and CH compiled and submitted all documentation for IRB ethics review and OSF pre-registration. AB and AC oversaw data collection and analysis. AB wrote the first draft of the manuscript. All authors collectively contributed to the conception and design of the study and assisted with subsequent revisions.

## Conflict of Interest

The authors declare that the research was conducted in the absence of any commercial or financial relationships that could be construed as a potential conflict of interest.

## Footnotes

^1^We note that intergroup relations were also a salient theme in the 2020 election (e.g., the role of the Black Lives Matter movement); however, as our data were collected in 2017, we emphasize the 2016 election in this paper.

^2^Though a majority of all non-White voters supported Hillary Clinton over Donald Trump, the exit polls showed that the greatest differential was among Black voters, who voted in Clinton's favor by a margin of 89 to 8% (CNN, 2016). Thus, we chose to use Black voters as a comparison group to the Caucasian sample.

^3^The Pearson correlation between national nostalgia and outgroup prejudice reported by Smeekes and Verkuyten, 2015, study 2).

^4^The authors would like to note that this scale was not included in the original pre-registration, as it was published just prior to the time this study was developed. However, the decision was made prior to data collection to utilize this validated scale as a more direct and statistically sound way to measure the construct of national nostalgia.

^5^Although structural equation models are often used to model paths among composite variables (such as national and personal nostalgia), we opted to use a path model for these analyses given that our sample was not large enough to justify inclusion of all individual items in the model.

^6^Although RMSEA greater than 0.08 is often considered marginal fit, RMSEA has been known to become inflated with sample sizes lower than 200 (Meyers et al., 2016).
